# Male breast cancer: 22 case reports at the National Hospital of Niamey-Niger (West Africa)

**DOI:** 10.4314/pamj.v3i1.52454

**Published:** 2009-11-16

**Authors:** Sani Rachid, Harouna Yacouba, Nouhou Hassane

**Affiliations:** 1 Department of Surgery, National Hospital of Niamey. PB: 238 – Niamey – Niger –Tel: 00 227 20 72 22 53,; 2 Department of histopathology-Faculty of Medicine. PB: 10896 – Niamey, Niger

**Keywords:** Male breast cancer, treatment, Niger

## Abstract

**Background:**

Male breast cancer (MBC) is rare. The objective of the study is to report clinicopathological characteristics, treatment patterns, and outcomes of MBC.

**Method:**

This study, which includes two parts (retrospective and prospective), focused on all hospitalized male patients with breast cancer during 17 years (1992–2008) with histological confirmation.

**Results:**

The series included 22 patients. The mean age was 52.8 years (range: 28–80 years). MBC represented 5.7% of all breast cancers. Most patients had an advanced disease with skin ulceration and inflammation T3 (31.9%) and T4 (59.1%). The majority of patients came from rural areas (63.6%). The duration of signs ranged from 1 to 7 years. Histology found infiltrating ductal carcinoma in 14 cases (63.6%), sarcoma in 3 cases (13.6%), papillary carcinoma in 2 cases (9%), and lobular carcinoma, medullar carcinoma, and mucinous carcinoma in 4.6% each of the others cases. The treatment had consisted of a radical mastectomy (Halsted or Patey) in 19 cases (86.4%) with axillary clearance and incomplete resection in 3 cases (13.6%). In the retrospective study follow-up of 14 patients, we lost sight of 13 patients 6 months after surgery. In the prospective study of 8 patients 10 to 36 months after mastectomy, 4 patients were deceased (50%), 4 were alive with 1 case having a local recurrence and pulmonary metastasis.

**Conclusion:**

The advanced clinical forms of MBC are most frequent with skin ulceration and nodal enlargement. The absence of radiotherapy and the low access of chemotherapy limited the treatment to radical mastectomy (Halsted) in the majority of cases.

## Background

The clinical and histological criteria of male breast cancer (MBC) are well defined. In developed countries, MBC accounts for 1–5.7% of breast cancer and 0.2–1.5% of all cases of malignancies in males [1–5]. The causes of MBC remain unknown; however several risk factors exist. The scarcity of this pathology explains the absence of clinical and therapeutic exploratory studies. As a result, MBC has not been studied as extensively as female breast cancer. Most studies related to MBC are retrospective analyses with a small number of patients; carcinoma is the most frequent histological type and the treatment of MBC is patterned on that of the females [2, 5]. The appearance of the cutaneous signs is the most frequent reason for consultation. Because of ignorance and/or negligence on the part of patients, they often seek medical treatment late. In addition, ignorance of MBC is sometimes found in certain medical staff which leads to wrong decision and management of these cancers, in the context of Niger where the diagnostic and therapeutic means are often unavailable or inaccessible to patients.

The clinicopathological characteristics, treatment patterns and outcomes of MBC were investigated in this study at the National Hospital of Niamey (NHN) which is the highest reference center in Niger.

## Method

This study covers 17 years, with a retrospective part for 14 years (January 1992–December 2005) and a prospective study for 3 years (January 2006–December 2008). The study was carried out at the National Hospital of Niamey and the department of histopathology of the Medical College of Niamey which keeps the National Registry of Cancer of Niger. Included in the study were all patients with MBC with histological confirmation. Data were collected from the files of the Department of Surgery and the National Registry of Cancer book. The studied variables were data regarding general characteristics of patients (age, presenting signs and symptoms, duration of symptoms, and site and location of tumor), histopathology of tumors, TNM and UICC staging, treatment modalities (surgery, chemotherapy, radiation and hormone therapy), and survival.

## Results

### General characteristics of patients

The study included 22 patients: 14 cases for the retrospective study and 8 for the prospective study. A total of 383 cases of breast cancer had histopathological confirmation, of whom 361 cases were female (94.3%) and 22 cases male (5.7%). The frequency of the MBC was 1.3 cases per year. The average age was 52.8 years (range: 28–80 years). The risk factors found are represented by the gynaecomastia in 3 cases (13.6%), breast trauma, jaundice; obesity and sterility in 1 case each (4.6%). The majority of patients came from rural areas in 14 cases (63.6%) and from Niamey in 8 cases (36.4%).

### Clinical features

**Figure 1:**
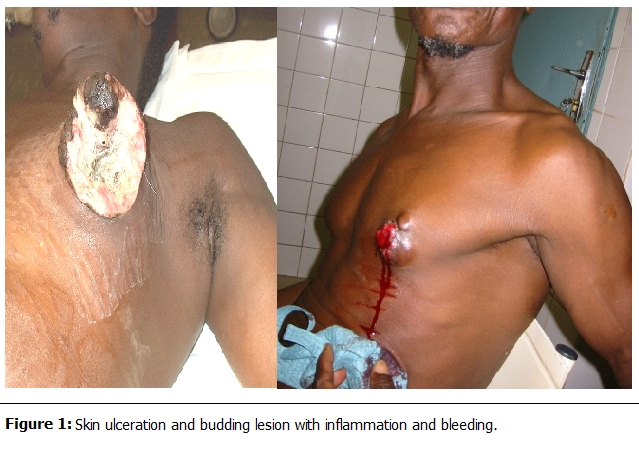
Skin ulceration and budding lesion with inflammation and bleeding

**Figure 2:**
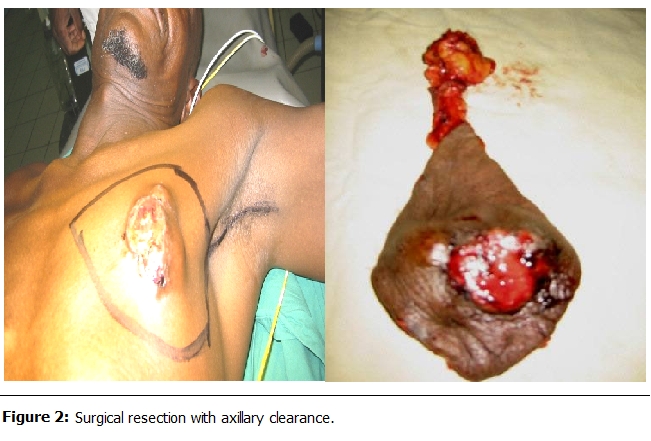
Surgical resection with axillary clearance

The most frequent clinical presentation was skin ulceration and hemorrhagic budding type ( Figure 1 and Figure 2 ) in 14 cases (63.6%), followed by 8 cases (36.4%) of retro-areolar nodule (with inflammatory tumefactions in 3 cases). The duration of the evolution of these signs (time passed between the discovery of the tumor and the first consultation) varied from 1 to 7 years. The clinical examination found palpable lymph nodes in the axillary region in 15 cases (68.2%). The tumor was found on the left breast in 15 cases (68.2%) and on the right breast in 7 cases (31.8%); there was no case of bilateralism. Investigations to assess the extend of the disease have shown three cases (13.6%) of synchronous pulmonary metastases.

### Histopathological characteristics of tumor

The majority of cases were classified T3 and T4 with 20 cases (91%) ( Table 1 ) The results of the pathological examination found infiltrating ductal carcinoma in 14 cases (63.6%), sarcoma in 3 cases (13.6%), papillary carcinoma in 2 cases (9%), and in the others cases 4.6% each of lobular carcinoma, medullar carcinoma and mucinous carcinoma. The hormonal receptor was carried out in 3 patients (13.6%); one was positive and two were negative. Among the 15 cases of node resection 10 cases were positive pN+ (66.7%), 4 were negative pN-(26.6%), and 1 case (6.7%) was unspecified. The cutaneous invasion was found in 12 cases (54.5%).

### Treatment patterns and survival

The treatment was essentially surgical ( Table 2 ). The surgery consisted of a modified radical mastectomy (PATEY n= 5) or according to HALSTED (n=14) in 19 cases (86.7%) with axillary clearance ( Figure 2 ). In three cases (13.6%) an incomplete resection was performed. No patients received neoadjuvant chemotherapy. After completion of surgery, adjuvant therapies were administered and four patients received chemotherapy (cyclophosphamide, fluorouracil). One patient (4.6%) received hormone therapy with Tamoxifen.

In the retrospective study (n=14 patients): 13 patients (92.8%) were lost in sight 6 months after surgery and 1 death (7.2%) was recorded in the 13th month after mastectomy. In the prospective study (8 patients) after a back throw of 10 to 36 months, 4 deaths (8th, 10 ^th^ , 14 ^th^ and 18th month) (50%) were recorded, 4 patients were alive with 1 case presenting a local recurrence and pulmonary metastasis.

## Discussion

MBC is a rare disease, accounting for 5.7% of all BC in our study. However, the incidence of MBC has been increasing significantly along with the increasing incidence of female breast cancer, although geographic variations in the incidence of MBC were reported. In Europe, approximately 1% of all BC occurs in males, but the incidence is much higher in other areas such as sub-Saharan Africa with 5 to 15% [4–6]. According to Simon et al. [7] these differences do not have any racial basis. The mean age at the time of the diagnosis is of 52.8 years, approximately one decade more than that of females in Niger (35–44 years) [8]. The bimodal age distribution seen in women is absent in men; the incidence increases exponentially with age [2].

MBC in Western countries was presented mostly in men in their 60s (range: 63–68 years), which is 10 years later than in females. MBC is rare before the age of 40 years [1–6]. The study of the aethiopathogenic factors of MBC is unclear; however many risk factors have been suggested. Among these factors, some are common in men and women: chest wall irradiation, breast trauma and endogenous hyperoestrogenism secondary to a hepatic dysfunction due to a parasitic disease (bilharzias) or the viral infection (hepatitis B) what would explain the high frequency in Africa and Asia and gynaecomastia [3–5, 9]. Other risk factors include Klinefelter syndrome, obesity, alcohol consumption, and gene deteriorations [2–4, 6, 10]. In our study the risk factors found were gynaecomastia (13.6%), breast trauma (4.6%), jaundice (4.6%), and obesity (4.6%). The clinical feature is dominated by the skin ulcero-hemorrhagic lesion (63.6%), this sign is accounted for 52–73.1% in African studies [3, 11–13].

TNM staging finds a high distribution of T3 and T4 (91%) in this study, which means an advanced stage of cancer. More than 40% of patients with MBC present with stage III or IV disease in Western countries, while in Africa the rate varies from 54–100% [2, 3, 5, 12–15]. Several reasons can account for the high distribution in Africa including ignorance of the patients, error in initial diagnostic in rural health centers where the first consultations are conducted by nonmedical personnel. Because of these factors, MBC is often confused with benign pathologies of the skin. This ignorance, associated with the low economic levels of the population, means that most patients initially turn to traditional medicine and the hospital is generally the last remedy. The duration of the evolution of MBC in Africa is usually longer, which explains the advanced clinical presentation: it varies from 1 to 7 years in our study. According to El Hajjam et al [3] this duration varies from 4 to 36 months. Infiltrating ductal carcinoma is the most frequent invasive carcinoma in men, accounting for 70–95% of MBC, while lobular carcinoma is rare (around 1% of all cases) due to lack of terminal lobules in the male breast. The rarer subtypes, such as carcinomas (medullary, tubular, mucinous, and squamous) and sarcoma, have all been reported in men, although they may be slightly more uncommon than in women [1–6, 14]. The node metastasis (pN+) is of 66.7% among 13 patients which underwent axillary clearance; in the literature the rate varies from 35 to 84% [3–5, 12]. The hormone-dependence of MBC is established, the hormonal receptors are positive in 65 to 90% of cases according to the series, those with estrogens in 65 to 86% and those with progesterone in 67 to 80% [4, 16]. In our study these receptors were tested in three patients and one was positive (33.3%). Because laboratories in Niger do not have the equipment to carry out this type of investigation, these three cases were done in Germany.

The treatment guideline has been extrapolated from the data based on female breast cancer: surgery, radiotherapy chemotherapy, and hormone therapy. Currently the modified radical mastectomy supplanted the radical mastectomy with comparable carcinological results for tumors seen precociously for certain authors [2, 3, 5, 17]; Crichlow [18] recommends the mastectomy according to Halsted procedure, the preservative methods in application in women should not be used in men (central tumors, invasion the skin, and the pectoral muscle). In our study where the tumors are locally advanced the mastectomy according to Halsted was carried out in the majority of the cases. The radiotherapy was carried out in one case (4.6%) in this study, it is indicated in the presence of risk factors of local recurrence (metastatic lymph node, cutaneous invasion, reduced safety margins); Several studies have found that radiation reduces the risk for local recurrence but does not change the overall survival [2, 4]. The creation of the service of oncology 3 years ago has made chemotherapy possible for 18.2% of patients; chemotherapy became a standard treatment using several protocols comparable with those of women [5, 10]. The neo-adjuvant chemotherapy of induction allows for early treatment of the systemic disease and for the reduction of tumoral volume with sometimes complete response, it is indicated in advanced T2, T3, and inflammatory cancers T4 [5, 10]. Because of high expression rates of hormone receptor positivity in MBC, adjuvant hormone therapy with Tamoxifen is theoretically the rational therapeutic strategy and should be considered in men with BC [2]. Most authors showed that the adjuvant hormone-therapy improved the survival rate and the rate of remission supplements and recommend a systematic hormone therapy for all the N1 stages and metastases [2, 5, 10]. MBC patients seem to have a less positive prognosis than female breast cancer patients due to a later discovery and a more advanced stage in men. A comparison between men and women with same stage indicate any difference in their prognosis [4–6, 10]. The two independent prognostic factors are tumor size (which has also been shown to be a significant prognostic factor) and lymph node invasion. The 5-year overall survival rates for all stages of breast cancer in men have been reported to range from 36% to 66%, and 10-year overall survival rates range from 17% to 52% [1, 2, 16]. In our environment where the optimal therapeutic conditions are not met, the prognosis remains very poor with 50% of death before 2 years. All the patients that were lost of sight in the retrospective study (n= 13 patients) would have probably died in anonymity in a regional or a rural hospital where there is no reliable filing service.

## Conclusion

MBC is a rare affection, still ignored in our environment. MBC has the same clinical and histopathological characteristics as female breast cancer. The advanced clinical forms are most frequent in our environment. The absence of a center of radiotherapy and the inaccessibility of chemotherapy have limited the treatment to surgery (radical mastectomy), associated with axillary dissection. Therefore, education, an appropriate system for early detection, and adequate treatment are necessary for improving outcomes.

## Figures and Tables

**Table 1:** Patients with breast cancer according to International union Against Cancer (UICC) stages and TNM classification

** UICC Stages **	** TNM **	** Numbers **	** Total **
** I **		0	0
** II **	T2N0M0	1	2(9%)
	T2N1M0	1	
** III **	T3N0M0	2	7 (31.9%)
	T3N1M0	4	
	T3N2MX	1	
** IV **	T4N0M0	1	13 (59.1
	T4N1M0	6	
	T4bN1M0	1	
	T4N2M0	2	
	T4N1M1	3	
** Total **			** 22 (100%) **

**Table 2:** Treatment of patients with breast cancer at the National Hospital of Niamey

** Types of treatment **	** Number **	** % **
Surgery only	17	77.2
Surgery and chemotherapy	3	13.6
Surgery + Chemotherapy+ Radiotherapy	1	4.6
Surgery + Hormone therapy	1	4.6
** Total **	** 22 **	** 100 **

## References

[1] Joli R, Weiss JR, Moysich KB, Swede H (2005). Epidemiology of Male Breast Cancer. Cancer Epidemiol Biomarkers Prev.

[2] Giordano SH, Buzdar AU, Hortobagyi GN (2002). Breast cancer in men. Ann Intern Med.

[3] El Hajjam M, Khaiz D, Benider A, Abi F, Kahlain A, Bouzidi A (1995). Male Breast Cancer, 50 case reports. J Chir (Paris).

[4] Benchellah Z, Wagner A, Harchaoui Y, Huten N, Body G (2002). Male Breast Cancer, 19 case reports. Ann Chir.

[5] Park S, Kim JH, Koo J, Park BW, Lee KS (2008). Clinicopathological characteristics of male breast cancer. Yonsei Med J.

[6] Ben Dhiab T, Bouzid T, Gamoudi A, Ben Hassouna J (2005). Male breast cancer. Bull Cancer.

[7] Simon MS, McKnight E, Schwartz A, Schwartz A, Martino S, Swanson GM (1992). Racial differences in cancer of the male breast; 15 years experience in the Detroit Metropolitan area. Breast Cancer Res. Treat.

[8] Nayama M, Nouhou H, Souna-Madougou K, Idi N, Garba M, Tahirou A, Toure A (2006). Epidemiological and histological aspects of gynecologic and breast cancer in histopathologic department of Niamey’s health faculty (Niger). Mali Medical.

[9] Beyrouti M, Kharrat Koubaa M, Affes N, Ben Ali I, Abbes I, Frikha M, Daoud J, Kechaou M, Jlidi R (2003). Male breast cancer. Tunisie Médicale.

[10] Nahleh ZA, Srikantiah R, Safa M, Jazieh Ar, Muhleman A, Komrokji R (2007). Male breast cancer in the veterans’ affairs population: a comparative analysis. Cancer.

[11] Kidmas AT, Ugwu BT, Manasseh AN, Iya D, Opaluwa AS (2005). Male breast malignancy in Jos University Teaching Hospital. West Afr J Med.

[12] Maalej M, Frikha H, Ben Salem S, Daoud J, Bouaouina N, Ben Abdallah M (1999). Breast cancer in Tunisia. Bull Cancer.

[13] Sano D, Dao B, Lankoande J, Toure B, Sakande B, Traore S (1997). Male breast cancer in Africa, Apropos of 5 cases at the Ouagadougou University Teaching Hospital (Burkina Faso). Bull Cancer.

[14] Oguntola AS, Aderonmu AO, Adeoti Ml, Olatoke SA, Akanbi O, Agodirin SO (2009). Male Breast Cancer in LAUTECH Teaching Hospital Osogbo, South Western Nigeria. Niger Postgrad Med J.

[15] Heller KS (1978). Male breast cancer: a clinicopathologic study of 97 cases. Ann Surg.

[16] Adami HO, Holmberg L, Malker B, Ries L (1985). Long-term survival in 406 males with breast cancer. Br J Cancer.

[17] Goss PE, Reid C, Pintilie M, Lim R, Miller N (1999). Male breast carcinoma: a review of 229 patients who presented to the Princess Margaret Hospital during 40 years: (1955–1996). Cancer.

[18] Crichlow R, Galt SW (1990). Male breast cancer. Surg Clin North Am.

